# Investigation of Hemoglobin/Gold Nanoparticle Heterolayer on Micro-Gap for Electrochemical Biosensor Application

**DOI:** 10.3390/s16050660

**Published:** 2016-05-09

**Authors:** Taek Lee, Tae-Hyung Kim, Jinho Yoon, Yong-Ho Chung, Ji Young Lee, Jeong-Woo Choi

**Affiliations:** 1 Department of Chemical and Biomolecular Engineering, Sogang University, 35 Baekbeom-ro (Sinsu-dong), Mapo-gu, Seoul 121-742, Korea; nanotlee@gmail.com (T.L.); iverson@naver.com (J.Y.); jylee72@sogang.ac.kr (J.Y.L.); 2 Research Center for Integrated Biotechnology, Sogang University, 35 Baekbeom-ro (Sinsu-dong), Mapo-gu, Seoul 121-742, Korea; 3 School of Integrative Engineering, Chung-Ang University, Heukseok-dong, Dongjak-gu, Seoul 156-756, Korea; thkim0512@gmail.com; 4 Department of Chemical Engineering, Hoseo University, 20, Hoseo-ro 79beon-gil, Baebang-Eup, Asan City, Chungnam 336-795, Korea; yhchung@hoseo.edu

**Keywords:** electrochemical biosensor, hemoglobin, gold nanoparticle, cyclic voltammetry, Au micro-gap

## Abstract

In the present study, we fabricated a hemoglobin/gold nanoparticle (Hb/GNP) heterolayer immobilized on the Au micro-gap to confirm H_2_O_2_ detection with a signal-enhancement effect. The hemoglobin which contained the heme group catalyzed the reduction of H_2_O_2_. To facilitate the electron transfer between hemoglobin and Au micro-gap electrode, a gold nanoparticle was introduced. The Au micro-gap electrode that has gap size of 5 µm was fabricated by conventional photolithographic technique to locate working and counter electrodes oppositely in a single chip for the signal sensitivity and reliability. The hemoglobin was self-assembled onto the Au surface via chemical linker 6-mercaptohexanoic acid (6-MHA). Then, the gold nanoparticles were adsorbed onto hemoglobin/6-MHA heterolayers by the layer-by-layer (LbL) method. The fabrication of the Hb/GNP heterolayer was confirmed by atomic force microscopy (AFM) and surface-enhanced Raman spectroscopy (SERS). The redox property and H_2_O_2_ detection of Hb/GNP on the micro-gap electrode was investigated by a cyclic voltammetry (CV) experiment. Taken together, the present results show that the electrochemical signal-enhancement effect of a hemoglobin/nanoparticle heterolayer was well confirmed on the micro-scale electrode for biosensor applications.

## 1. Introduction

In the field of nanobiotechnology, the electrochemical study of biomolecules plays an important role in understanding the electron transfer mechanism of living organisms [1,2]. Recently, the electrochemical study of biomolecules has made advances in the form of nano-biotechnologies, such as the fabrication of biochips, biosensors, and bioelectronic applications [3,4]. The electrochemical analysis of biomolecules not only provides an understanding of their electrochemical behavior, but also furthers applications to biomedical and environmental biosensors. In particular, an electrochemical-based biosensor composed of protein can be a very effective tool to characterize a target analyte [5]. Various types of electrochemical-based biosensor composed of protein such as antibody and enzyme have been reported for environmental, biomedical, and food industry applications [6,7,8,9].

In particular, the detection of hydrogen peroxide (H_2_O_2_) is a quite significant chemical compound in the clinical, environmental, pharmaceutical, and cosmetic industries [10,11]. Within a living organism H_2_O_2_ is a reactive oxygen by-product which related with key elements as a regulating diverse biological stress. The oxidative stress related with H_2_O_2_ has been linked to cytotoxic effects and immune cell activation, as well as intracellular thermogenesis [12]. For these reasons the detection of H_2_O_2_ at low concentration is considered to be an important analyte. Usually, various enzymes such as peroxidases, lipoxygenase, and catalases are used to detect H_2_O_2_ for biosensor applications [13,14,15]. However, these enzymes are too sensitive, unstable, and expensive. Recently, metalloproteins have been introduced to detect H_2_O_2_ because they can provide a direct transfer of electrons and improved sensitivity [16].

Hemoglobin (Hb) is a well-investigated metalloprotein with a molecular weight of ~64.5 kDa, and has four electroactive ferrous ions (Fe^2+^) in a heme group that are oxidized to ferric ions (Fe^3+^). It has been studied in the context of the life sciences, pharmaceutical sciences, and medicine [17,18]. In living organisms, hemoglobin transports oxygen in red blood cells [12]. Recently, hemoglobin has been used to develop an enzymeless H_2_O_2_ biosensor for peroxidase activity. Because the hemoglobin provides the direct electron transfer (DET) to achieve the fast electron transfer with high sensitivity [19,20].

Several groups have studied the hemoglobin-based biosensor on a conventional electrode surface [12,21,22,23,24,25], while others introduced nanoparticles [21], graphene [22] and other heavy metal materials [23] on an electrode to facilitate the electron transfer between H_2_O_2_ and hemoglobin. However, such heavy metal-based nanomaterials are toxic and possess low biocompatibility. A gold nanoparticle was introduced to facilitate the electron transfer between hemoglobin and Au electrode. The gold nanoparticle is toxic at low levels and widely used to fabricate the electrochemical biosensor [26,27]. Furthermore, the electrochemical-based biosensor has been studied on a bulk scale electrode. Some groups proposed miniaturized biosensor to detect the ErbB2 protein [28], PSA [29] and DNA hybridization [30]. Those miniaturized biosensors provide (1) defined distance between working and counter electrodes; (2) detect the low concentration of sample. To develop the advanced electrochemical biosensor based on direct electron transfer, the miniaturized biosensor with high sensitivity will be needed.

**Figure 1:**
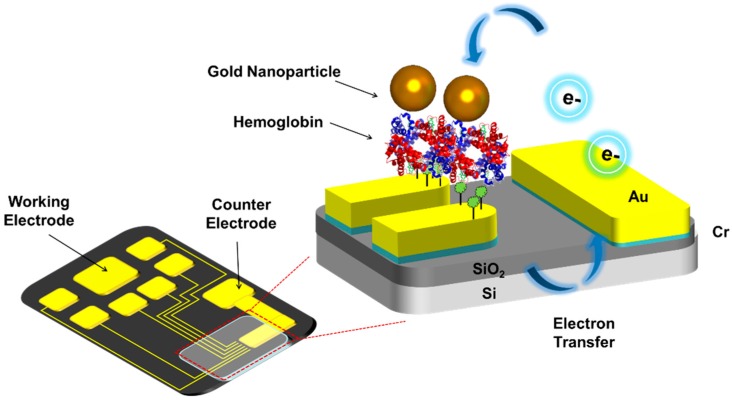
Schematic diagram of fabricated hemoglobin/gold nanoparticle heterolayer immobilized on the micro-gap for H_2_O_2_ detection.

In the present study, the hemoglobin/gold nanoparticle heterolayer was fabricated on the micro-gap for signal-enhanced electrochemical H_2_O_2_ biosensor applications. To determine the H_2_O_2_ detection property, the hemoglobin/gold nanoparticle (Hb/GNP) was immobilized onto the Au electrode and Au micro-gap electrode through self-assembly method. The immobilization of the hemoglobin/gold nanoparticle heterolayer was confirmed by atomic force microscopy (AFM) and surface-enhanced Raman spectroscopy (SERS). The performance of the hemoglobin/gold nanoparticle heterolayer biosensor was investigated by cyclic voltammetry (CV). Figure 1 shows the schematic diagram of fabricated hemoglobin/gold nanoparticle immobilized on the Au micro-gap.

## 2. Materials and Methods

### 2.1. Materials

The Au substrates (Au (43 nm)/Cr (2 nm)/SiO_2_ (200 nm) Si (p-type) wafers) were prepared to carry out the AFM and CV experiments to manufacture the working electrode (G-MEK, Seoul, Korea). Negative photoresist (Su-82), developer, and stripper for the photolithographic process were purchased from Microchem (Westborough, MA, USA). Pt wire for the counter electrode and the Ag/AgCl reference electrode were purchased from BAS (West Lafayette, IN, USA) to conduct the electrochemical experiments. The 6-Mercaptohexanoic acid (6-MHA) and hemoglobin extracted from *horse heart* were purchased from Sigma-Aldrich (Saint Louis, MO, USA). A 0.2 mg/mL hemoglobin solution was prepared and dilluted in 10 mM HEPES buffer at pH 7.2. For the electrochemical experiment, hydrogen peroxide (H_2_O_2_) was purchased from Daejung Chemical and Metals Co. Ltd. (Siheung-si, Korea). The gold nanoparticles (20 nm and 60 nm) were purchased form BBI for electrochemical experiment and SERS experiment, respectively (Cardiff, UK).

### 2.2. Fabrication of Micro-Gap

The Si substrate (100) with a thermally oxidized SiO_2_ was initially cleaned with piranha solution composed of H_2_SO_4_ (Daejung Chemical and Metals Co. Ltd., Siheung-si, Korea) and H_2_O_2_ (Duksan Pure Chemical and Metals Co. Ltd., Seoul, Korea) with a volume ratio of 5:1 at 70 °C for 10 min, and baked on the hot plate at 200 °C for 20 min for adhesion promotion with photoresist. The pre-pattern for metal deposition was fabricated by the standard photolithographic process using negative photoresist. Initially, Su-82 photoresist was spin-coated in cleaned substrate with a speed of 3000 rpm, and then baked at 65 °C for 1 min and 95 °C for 1 min. After exposure of 110 mJ/cm^2^ (i-line) by MA6 mask aligner (SUSS MicroTec., Garching, Germany), substrate was post-baked by the same steps with pre-baking and developed for 70 s. Metal deposition of Cr (5 nm) and Au (50 nm) was operated with an electron beam evaporator. Finally, a lift-off process using remover PG was performed at 70 °C for 2 h. The fabricated substrates were cleaned by piranha solution with the same conditions. The working chamber, which has the volume of 400 μL for electrochemical measurements, was attached on the prepared substrate by using PDMS linkage. After attachment of the chamber on the multi-electrode, substrates were immersed in acetone and isopropyl alcohol (IPA) for 20 min sequentially to remove organic contaminant, which could be induced in the curing process of PDMS at high-temperature chamber.

### 2.3. Fabrication of Hemoglobin/Gold Nanoparticle Heterolayer

**Figure 2:**
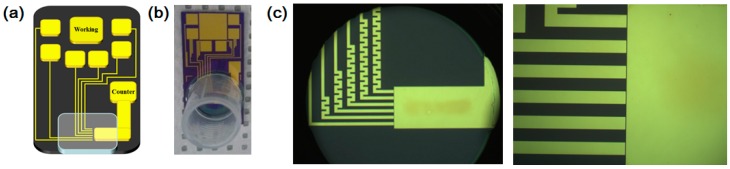
(**a**) Schematic diagram of micro-gap electrode; (**b**) Optical image of fabricated micro-gap electrode with working chamber for H_2_O_2_ biosensor application; (**c**) Optical image of zoomed micro-gap.

To fabricate a micro-gap-based electrochemical sensor, the prepared Au micro-gap was treated with piranha solution at 70 °C for 3 min, washed with deionized water, and dried under N_2_ gas to remove dust and residue before the self-assembly process, [31,32]. Then, 3 μL of 10 mM 6-MUA was added onto the Au surface for 3 h. The thiol group of the 6-MHA molecule was bridged as a terminal group to the Au surface by covalent bonding during the immobilization period. The remaining 6-MHA on the surface was rinsed thoroughly with DI water and ethanol to remove excess residue. Then, 5 μL of the 0.2 mg/mL hemoglobin solution was added onto the self-assembled 6-MHA layer over 6 h. During this step, the free amine group of hemoglobin was bound to the carboxyl groups of 6-MHA by EDC/NHS reaction. Additionally, the modified substrates were cleaned with deionized water and dried under an Ar gas stream. Then, 50 mM solution of 1-Octadecanethiol was added for 6 h, and the prepared 0.2 mg/mL of gold nanoparticle solution (3 μL) was added onto the hemoglobin self-assembled substrate for 6 h. Finally, the modified substrates were cleaned with deionized water and dried under an Ar gas stream [33]. Figure 2a–c show the schematic diagram, the optical image of fabricated micro-gap electrode with working chamber for H_2_O_2_ and optical image of zoomed Au micro-gap electrode, respectively.

### 2.4. Investigation of Hemoglobin/Gold Nanoparticle Heterolayer Using AFM and SERS

To confirm the immobilization of the Hb/GNP hybrid layer, the surface topography of the Hb/GNP heterolayer was investigated through AFM. The tapping-mode AFM was operated with a Nanoscope IV/Multimode from Digital Instruments. The tips used for the AFM measurement were phosphorous (n-type doped Si), and the resonance peaks in the frequency response of the cantilever were selected at around of 230–305 kHz. Spring constants of 20–80 N/m were used. Before scanning the sample, the set point was adjusted to optimize the force between the tip and the biomolecule.

For SERS measurement, indium tin oxide (ITO)-coated glass (G-mek, Seoul, Korea) was prepared by sonication for 30 min in 1% Triton X-100, DIW, and ethanol, sequentially. The preparation of SERS substrate was followed by our previous study. Then, the hemoglobin was immobilized via 6-MHA linker, and the gold nanoparticle was dropped onto the hemoglobin-modified substrate for SERS measurement [34].

### 2.5. Electrochemical Analysis of Hemoglobin-Gold Nanoparticle Heterolayer

An electrochemical H_2_O_2_ detection test of the Hb/GNP heterolayer was performed using a CHI660A electrochemical workstation (CH Instruments, Austin, TX, USA) and a three-electrode system. The Hb/GNP immobilized on the working electrode in the micro-gap. And the counter electrode part in micro-gap was used as a counter electrode and an Ag/AgCl (saturated KCl) electrode was used for a reference electrode. The electrochemical analysis for the H_2_O_2_ sensitivity was performed in 10 mM PBS buffer solution (pH = 7.4) at room temperature [16]. The electrochemical experiment was repeated with five samples.

## 3. Results and Discussion

### 3.1. The Surface Investigation of Hemoglobin/Gold Nanoparticle Hybrid by AFM and SERS

**Figure 3:**
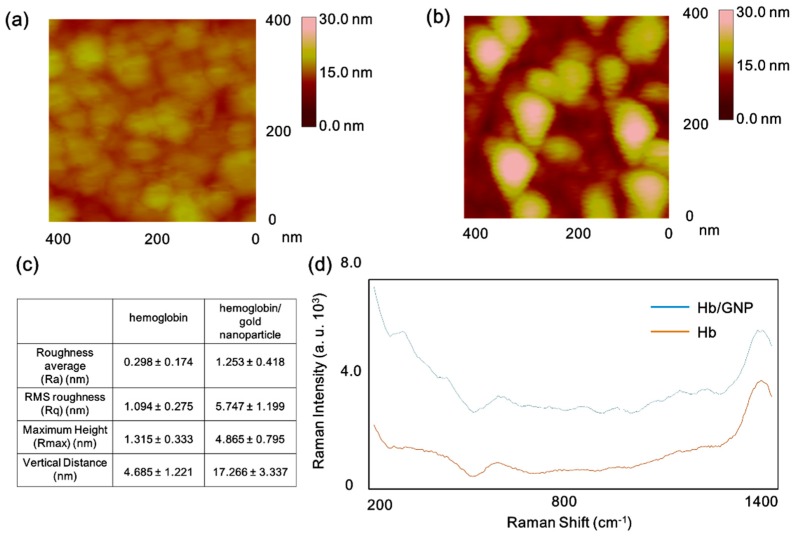
Surface morphology investigation of (**a**) hemoglobin; (**b**) hemoglobin/gold nanoparticle on 6-mercaptohexanoic acid (6-MHA) layer; (**c**) Surface roughness analysis of the hemoglobin, hemoglobin/gold nanoparticle; (**d**) Raman spectra of hemoglobin (Brown line); (**b**) hemoglobin/gold nanoparticle on 6-MHA layer (Blue line).

AFM measurement was conducted to investigate the surface morphology of fabricated Hb/GNP heterolayer. The Hb self-assembled on 6-MHA layer and Hb/GNP hybrid layer were investigated. In the case of the hemoglobin surface, small lumps of protein clusters were oriented onto the Au substrate via 6-MHA linker. The average lump sizes were around 20–30 nm and the vertical distance of each lump was 4–7 nm (Figure 3a). However, the surface of the Hb/GNP heterolayer, the lump size, and vertical distance showed a different size and shape compared to hemoglobin’s lump. The size of the hemoglobin lumps were around 40–55 nm and vertical size around 15–20 nm (Figure 3b). The hemoglobin/gold nanoparticle clusters were larger than the lumps of Hb. Presumably, the result showed the gold nanoparticle was adsorbed on the hemolgobin molecule because the hemoglobin molecule was self-assembled onto the 6-MHA-modified Au substrate. As a result, the hemoglobin/gold nanoparticle was well oriented onto the Au substrate.

Moreover, the surface roughness of the Hb/GNP and Hb were analyzed using AFM Nanoscope software. The surface roughness values of the the Hb self-assembled on 6-MHA layer and the Hb/GNP self-assembled on 6-MHA layer are shown in Figure 3c, respectively. In the case of the Hb, the R_a_ value is 0.298 ± 0.174 nm, RMS roughness (R_q_) shows 1.094 ± 0.275 nm, and R_max_ is 1.315 ± 0.333 nm. Also, the average vertical distance of hemoglobin is 4.685 ± 1.221 nm. In the case of the hemoglobin/gold nanoparticle, the average R_a_, R_q_, R_max_ and vertical distance values are 1.253 ± 0.418 nm, 5.747 ± 1.199 nm, 4.865 ± 0.795 nm, and 17.206 ± 3.337 nm, respectively. Based on these results, the Hb/GNP heterolayer was well immobilized to the Au surface for further experiment.

Figure 3d displays the SERS spectra of the hemoglobin layer (Brown line) and the Hb/GNP heterolayer (Blue line), respectively. The Raman peaks of the Hb were monitored near the region of 1126 cm^−1^, 1214 cm^−1^; the 1126 cm^−1^ peak is assigned to the pyrrole half-ring stretching mode [35]. The intense peak at 1216 cm^−1^ was attributed to the C_m_H out-of-plane deformation stretching mode. And the Raman spectra of Hb was recorded near 651 cm^−1^, which had peaks assigned to the C-S (cys) stretching. The intense peak monitored near 1347 and 1356 cm^−1^ correspond to the anti-symmetric υ(pyr half-ring)_sym_. And the intense band near the region of 924 cm^−1^ was previously assigned to modes involving C-COO^−^ stretching [36]. Those peaks are monitored to both of SERS spectra. Compared to Hb, the Hb/GNP heterolayer possesses a greater SERS intensity. Based on this result, the Hb/GNP heterolayer was well-oriented onto the gold nanoparticle-coated ITO surface.

### 3.2. Cyclic Voltammetric Behavior of Hemoglobin/Gold Nanoparticle

**Figure 4:**
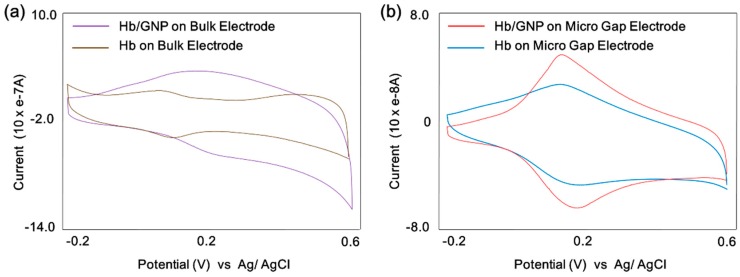
Cyclic voltammogram of (**a**) hemoglobin (Brown line) and hemoglobin/gold nanoparticle (Purple line) immobilized on bulk Au electrode, respectively; (**b**) hemoglobin (Red line) and hemoglobin/gold nanoparticle (Blue line) immobilized on micro-gap Au electrode, respectively.

A cyclic voltammetry was conducted to compare the redox properties of the Hb and Hb/GNP heterolayer-immobilized on a conventional Au electrode (1 × 2 cm) and Au micro-gap electrode. As shown in Figure 4a, the redox property of the Hb showed a quasi-reversible redox peaks (Brown line) because of the Fe^3+/2+^ redox center that provides the electron exchange. The anodic peak potential (*E_pa_*) and cathodic peak potential (*E_pc_*) were showed at 103 mV and 67 mV (*vs.* Ag/AgCl), respectively, with a peak-to-peak separation (Δ*E_p_*) of 36 mV. The redox property of the Hb/GNP showed a signal-enhanced redox peak (Purple line) because the GNP facilitates electron transfer between Hb and H_2_O_2_. The anodic peak potential (*E_pa_*) and cathodic peak potential (*E_pc_*) were 233 and 181 mV (*vs.* Ag/AgCl), respectively, with a peak-to-peak separation (Δ*E_p_*) of 52 mV. This result shows that the gold nanoparticles provide the higher surface roughness and active area which yields to a higher coverage [37].

Moreover, a CV test of Hb and Hb/GNP was carried out on the micro-gap electrode. The Figure 4b shows the cyclic voltamogram of Hb and Hb/GNP. In the case of Hb on the micro-gap electrode displayed a couple of well-defined quasi-reversible redox peaks (Blue line) because of the Fe^3+/2+^ redox center. The redox current signal was shown to be less than 10 times the bulk Au electrode, the anodic peak potential (*E_pa_*) and cathodic peak potential of Hb were determined to be 0.213 and 0.196 V (*vs.* Ag/AgCl) (Blue line) with a peak-to-peak separation (Δ*E_p_*) of 17 mV, which was narrow compared to the bulk Au electrode. The redox peaks of Hb/GNP showed a signal-enhanced redox peak (Red line) because the GNP facilitate electron transfer between Hb and H_2_O_2_ similar to the bulk Au electrode. The anodic peak potential (*E_pa_*) and cathodic peak potential (*E_pc_*) were 224 mV and 187 mV (*vs.* Ag/AgCl), respectively, with a peak-to-peak separation (Δ*E_p_*) of 37 mV, which was narrow compared to the bulk Au electrode. This result shows that the Hb/GNP heterolayer maintained the facilitation of the electron transfer on the micro-gap electrode.

### 3.3. Electrocatalytic Properties of Hemoglobin/Gold Nanoparticle on the Micro-Gap Electrode

**Figure 5:**
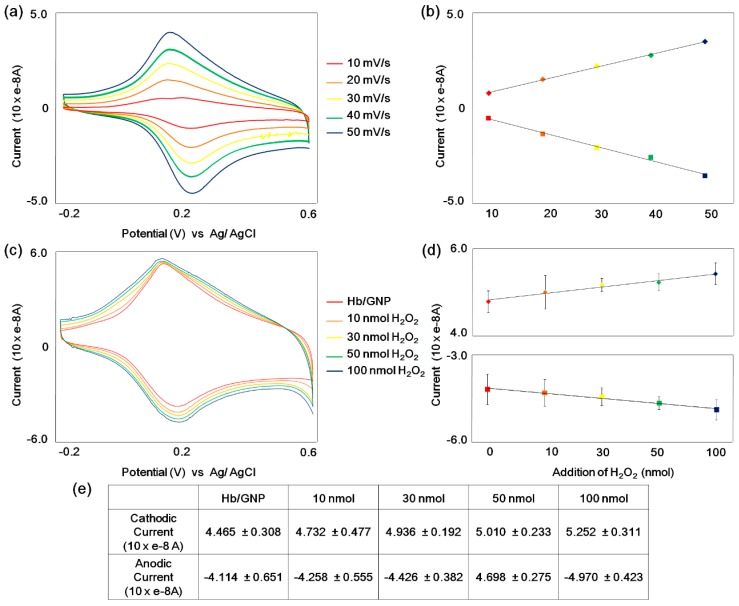
(**a**) Cyclic voltammogram of hemoglobin/gold nanoparticle (Blue line) immobilized on Au micro-gap electrode in 10 mM PBS (pH = 7.4) at different scan rate (mV/s) (Red line: 10 mV/s, Orange line: 20 mV/s, Yellow line: 30 mV/s, Green line: 40 mV/s, Blue line: 50 mV/s); (**b**) Plots of anodic and cathodic peaks currents *vs.* scan rates; (**c**) Cyclic voltammogram of hemoglobin/gold nanoparticle (Blue line) immobilized on Au micro-gap electrode containing (Red line: 0, Orange line: 10 nmol, Yellow line: 30 nmol, Green line: 50 nmol, Blue line: 100 nmol H_2_O_2_ at 50 mV/s); (**d**) Plots of anodic and cathodic peaks currents *vs.* addition of H_2_O_2_; (**e**) Table of current values corresponding to H_2_O_2_ concetrations.

Cyclic voltammograms of the Hb/GNP-modified micro-gap electrode were obtained with different scan rates from 0.01 to 0.05 V/s (Figure 5a). The result displays well-defined and slightly shifted cathodic and anodic peaks at each scan rate. Also, the CV result shows a linear plot for cathodic and anodic peak currents against the scan rate (Figure 5b), respectively. The linear regression equations were i_p_/10 × e^−8^ A = 0.0067 v(mV/s) − 0.0229 for cathodic currents and i_p_/10 × e^−8^ A = −0.0728 v(mV/s) − 0.0266 for anodic currents. The linearity of the CV results corresponded to the scan rate (v) show an increment of scan rate yields a slight shift of the cathodic peak to a more negative potential, while the anodic peak was moved to a more positive potential. Plotting E_pa_ and E_pc_ *vs.* log(scan rate) yielded two straight lines with a slope equal to −2.3 RT/αnF for the cathodic peak, and a slope of 2.3 RT/(1–α)nF for the anodic peak. The charge transfer coefficient was calculated (0.27) from the slopes of the straight lines based on the following equation [38]:

(1) $$\log\frac{k_{a}}{k_{c}}=\log\left[\frac{\alpha }{\left(1-\alpha \right)}\right]or\;\frac{k_{a}}{k_{c}}=\frac{\alpha }{1-\alpha }$$

where k_a_ is the slope of the line derived from E_pa_ = f(log scan rate); k_c_ is the slope of the line derived from; E_pc_ = f(log α).

The electron-transfer rate constant (*k_s_*) for electron transfer between the micro-gap electrode and the surface Hb layers was also 0.35 s^−1^ based on the following equation [16,38];

(2) $$\log k_{s}=\alpha \log(1-\alpha )+(1-\alpha )\log\alpha -\log\left(\frac{RT}{nF\nu }\right)-\frac{\alpha (1-\alpha )nF\Delta E_{p}}{2.3RT}$$

Also, the *Hb* shows electrocatalytic activity because of its similarity with peroxidase for catalysis of H_2_O_2_ [9,20]. The electrocatalytic activity of the Hb/GNP to H_2_O_2_ on the micro-gap electrode was investigated by CV. The principle of catalyzing H_2_O_2_ of Hb was explained using the following equations:

Hb[Fe(III)] + e^−^ → Hb[Fe(II)] (3)

2 [Hb − Fe(II)] + 2H^+^ + H_2_O_2_ → 2 [Hb − Fe(III)] + 2H_2_O (4)

The overall reaction would be:

H_2_O_2_ + 2H^+^ + 2e^−^ → 2 H_2_O (5)

Figure 5c shows the electrochemical signal-enhancement effect of anodic and cathodic currents corresponding to the successive addition of H_2_O_2_. The redox currents of Hb/GNP were gradually increased according to the addition of 10 nmol H_2_O_2_, 30 nmol H_2_O_2_, 50 nmol H_2_O_2_, 100 nmol H_2_O_2_. This result shows the electrocatalytic reduction process of the Hb/GNP heterolayer on the micro-gap. The anodic peak potential (*E_pa_*) and cathodic peak potential (*E_pc_*) were 0.207 and 0.205 V (*vs.* Ag/AgCl), respectively. As shown in Figure 5d,e, cathodic and anodic current peaks were obtained for the Hb/GNP heteroalyer on micro-gap electrode after the addition of 20 µL aliquots of 10 mM H_2_O_2_ in 1 mL of 10 mM HEPES at pH 7.0. The result shows a linear plot for anodic and cathodic peak currents against the addition of H_2_O_2_, and the sensor calibration plot was linear with R^2^ = 0.97 (anodic peak) and 0.98 (cathodic peak), respectively. The slope values of anodic and cathodic peak currents against the addition of H_2_O_2_, I_pa_ = 0.1862X + 4.3234(10 × e^−8^ A) and I_pc_ = −0.2152X − 3.8476(10 × e^−8^ A). And the 17.6% of cathodic peak current and 20.8% of anodic peak current were increased corresponding to the addition of H_2_O_2_. Based on this analysis, an increase of H_2_O_2_ concentration shifts the oxidation peaks to a more positive potential, while the reduction peaks shifted to a more negative potential on the micro-gap.

## 4. Conclusions

In summary, the Hb/GNP heterolayer on the micro-gap was fabricated to detect the H_2_O_2_ with electrochemical-signal enhanced effect. The fabricated Hb/GNP hybrid layer was investigated by AFM and SERS. Additionally, the redox properties of Hb/GNP hybrid layer on bulk electrode and micro-gap were confirmed using CV. Also, the H_2_O_2_ detection test of Hb/GNP heterolayer on the micro-gap was conducted. As a result, the fabricated Hb/GNP heterolayer on the Au micro-gap was detected the low concentration (10 nmol) of H_2_O_2_ with high fidelity. These results show the electrochemical signal-enhancement effect of Hb/GNP heterolayer can be directly applied to a protein-based biosensor system at the micro-scale. This metalloprotein-nanoparticle hybrid layer can be used for miniaturized H_2_O_2_ biosensor applications.

## Abbreviations

Hb	Hemoglobin
GNP	Gold Nanoparticle
AFM	Atomic Force Microscopy
SERS	Surface-Enhanced Raman Spectroscopy
6-MHA	6-Mercaptohexanoic Acid
LbL	layer-by-layer
CV	Cyclic Voltammetry
